# Automatic identification of human spermatozoa with zona pellucida-binding capability using deep learning

**DOI:** 10.1093/hropen/hoaf024

**Published:** 2025-05-10

**Authors:** Erica T Y Leung, Xianghan Mei, Brayden K M Lee, Kevin K W Lam, Cheuk-Lun Lee, Raymond H W Li, Ernest H Y Ng, William S B Yeung, Lequan Yu, Philip C N Chiu

**Affiliations:** Department of Obstetrics and Gynaecology, School of Clinical Medicine, Li Ka Shing Faculty of Medicine, The University of Hong Kong, Hong Kong, China; Department of Statistics and Actuarial Science, The University of Hong Kong, Hong Kong, China; Department of Obstetrics and Gynaecology, School of Clinical Medicine, Li Ka Shing Faculty of Medicine, The University of Hong Kong, Hong Kong, China; Department of Obstetrics and Gynaecology, School of Clinical Medicine, Li Ka Shing Faculty of Medicine, The University of Hong Kong, Hong Kong, China; Department of Obstetrics and Gynaecology, Queen Mary Hospital, Hong Kong, China; Department of Health Technology and Informatics, The Hong Kong Polytechnic University, Hong Kong, China; Department of Obstetrics and Gynaecology, School of Clinical Medicine, Li Ka Shing Faculty of Medicine, The University of Hong Kong, Hong Kong, China; Department of Obstetrics and Gynaecology, Queen Mary Hospital, Hong Kong, China; Department of Obstetrics and Gynaecology, School of Clinical Medicine, Li Ka Shing Faculty of Medicine, The University of Hong Kong, Hong Kong, China; Department of Obstetrics and Gynaecology, Queen Mary Hospital, Hong Kong, China; Department of Obstetrics and Gynaecology, School of Clinical Medicine, Li Ka Shing Faculty of Medicine, The University of Hong Kong, Hong Kong, China; Department of Obstetrics and Gynaecology, Queen Mary Hospital, Hong Kong, China; Hong Kong-Shenzhen Key Laboratory of Fertility Regulation, The University of Hong Kong-Shenzhen Hospital, Shenzhen, China; Department of Statistics and Actuarial Science, The University of Hong Kong, Hong Kong, China; Department of Obstetrics and Gynaecology, School of Clinical Medicine, Li Ka Shing Faculty of Medicine, The University of Hong Kong, Hong Kong, China; Hong Kong-Shenzhen Key Laboratory of Fertility Regulation, The University of Hong Kong-Shenzhen Hospital, Shenzhen, China

**Keywords:** human spermatozoa, conventional semen analysis, sperm morphology, zona pellucida-binding ability, Diff-Quik staining, IVF, ICSI, deep learning, automated identification, artificial intelligence

## Abstract

**STUDY QUESTION:**

Can a deep-learning algorithm, independent of World Health Organization (WHO) sperm morphology grading, be used to identify human spermatozoa with zona pellucida (ZP)-binding capability in assisted reproductive technology (ART)?

**SUMMARY ANSWER:**

A novel deep-learning model, irrespective of the conventional semen analysis, was established to identify human spermatozoa capable of binding to ZP for predicting their fertilization potential.

**WHAT IS KNOWN ALREADY:**

Sperm morphology evaluation is crucial in semen analysis to investigate male infertility and to determine the appropriate insemination methods in ART. The current manual assessment, which relies on microscopically examining individual spermatozoa based on WHO criteria, has shown limited predictive power for fertilization outcomes due to its highly subjective, labour-intensive nature, and high inter-/intra-assay variations. Deep learning is a rapidly evolving method for automated image analysis. Recent studies have explored its potential for automating sperm morphology analysis. However, algorithms trained on manually annotated datasets using existing WHO criteria have had little success in predicting ART outcomes. To date, no study has established an independent set of morphology evaluation standards based on sperm fertilizing ability for clinical prediction.

**STUDY DESIGN, SIZE, DURATION:**

Spare semen samples were collected from men undergoing premarital check-ups at a family planning clinic. Immature oocytes at germinal vesicle/metaphase I stage, or mature metaphase II oocytes were donated from women attending the infertility clinic for assisted reproduction treatments. Acrosome-intact, ZP-bound spermatozoa were collected by our previously modified spermatozoa-ZP coincubation assay. ZP-unbound spermatozoa were collected from normozoospermic samples with defective ZP-binding ability, as evidenced by complete fertilization failure following conventional *in vitro* fertilization (IVF) and the absence of ZP-bound spermatozoa on the inseminated oocytes. A total of 1083 Diff-Quik stained images of ZP-bound and unbound spermatozoa were collected to create a training database, with an additional 220 images serving as an independent test set. Clinical data were obtained from 117 men undergoing IVF due to male factor or unexplained infertility to validate the model’s ability to generalize to new data. These participants were categorized into three groups based on their fertilization rates following IVF: low (0–40%), intermediate (41–70%), and high (71–100%).

**PARTICIPANTS/MATERIALS, SETTING, METHODS:**

A pre-trained VGG13 model was fine-tuned using our database to classify individual spermatozoa as either ZP-bound or unbound based on their automatically extracted morphological features. Confusion matrix was used to assess the model’s classification performance, expressed in terms of accuracy, specificity, sensitivity, and precision rates. The area under the receiver-operating characteristic (ROC) curve (AUC) was utilized to measure the model’s discriminative power. A 5-fold cross-validation was conducted on the training dataset to assess the model’s performance on randomized subgroups. Saliency mapping was used to analyse pixel importance localized to the morphological features of sperm images. Clinical data of spermatozoa from three fertilization groups were used for clinical validation. Logistic ROC regression analysis was performed to evaluate the differences in predicted values between high and low fertilization groups, as indicated by AUC and *P*-values. Additionally, Youden’s index was applied to determine a clinical threshold for predicting IVF fertilization outcome using the model.

**MAIN RESULTS AND THE ROLE OF CHANCE:**

A VGG13 model was fine-tuned to distinguish images of spermatozoa capable of binding to the ZP based on their morphological features with high sensitivity (97.6%), specificity (96.0%), accuracy (96.7%), and precision (95.2%). The model exhibited low learning variance (average accuracy: 97.4%; sensitivity: 96.0%; and specificity: 98.5%) across subgroups, with primary emphasis on the sperm head and mid-pieces in all images as indicated by the pixel importance. Its discriminative performance was clinically validated on over 33 000 sperm images collected from three fertilization groups. Overall, the model exhibited excellent generalization ability as reflected by the strong correlation between the predicted percentages of spermatozoa with ZP-binding per sample and their fertilization rates. A clinical threshold of 4.9% (specificity: 89.3%; sensitivity: 90.0%) was established to differentiate sperm samples with normal and defective ZP-binding ability. By conducting pairwise comparisons among 30 patients, the predicted values generated by the model outperformed conventional semen analysis assessed by our in-house embryologists in identifying patients who were likely to experience failure with conventional IVF.

**LARGE SCALE DATA:**

N/A.

**LIMITATIONS, REASONS FOR CAUTION:**

The model is currently designed for high-resolution, air-dried, Diff-Quik stained sperm samples, and further research is required to validate its classification performance across different image qualities with a larger sample size.

**WIDER IMPLICATIONS OF THE FINDINGS:**

This newly established method can identify couples at high risk of unexpected IVF fertilization failure, enabling clinicians to offer alternative insemination methods to reduce the likelihood of suboptimal fertilization outcomes.

**STUDY FUNDING/COMPETING INTEREST(S):**

This study was supported in part by two Health and Medical Research Funds, the Food and Health Bureau, The Government of the HKSAR (07182446 and 11222236), and the Sanming Project of Medicine in Shenzhen (SZSM 202211014). Two provisional patent applications related to the data presented here have been filed on behalf of The University of Hong Kong (i. application no. 63/511,375; filing date: 30 June 2023; current status: active; applicant: The University of Hong Kong; ii. application no. US 63/567,147; filing date: 19 March 2024; current status: active; applicant: The University of Hong Kong). The authors declare that they have no other competing interests.

WHAT DOES THIS MEAN FOR PATIENTS? Sperm morphology evaluation is a highly subjective, labour-intensive approach to investigating male infertility in clinical settings. The method involves microscopic assessment of individual spermatozoa based on the World Health Organization (WHO) sperm morphology grading criteria, with limited predictive power for fertilization outcome following conventional *in vitro* fertilization (IVF). Deep learning is an advanced technique widely used for automated medical image analysis. It uncovers hidden but interconnected relationships within large datasets to make plausible predictions, surpassing conventional practices employed by human users. However, to date, none of the existing deep-learning models for sperm morphology evaluation have shown significant success in clinical prediction, due to their reliance on the use of manually annotated training data based on WHO criteria.

Therefore, we aimed to develop a novel deep-learning model to assess fertilization potential of human spermatozoa, as reflected by their capability of binding to the zona pellucida (ZP) surrounding human oocytes. We fine-tuned a deep-learning VGG13 model on a total of 1083 Diff-Quik stained images of ZP-bound and unbound spermatozoa, achieving exceptional classification performance with high sensitivity, specificity, accuracy, and precision rates. The model’s generalization ability was clinically validated on unseen clinical data of individually extracted sperm head images collected from 117 patients with male factor and unexplained infertility. There was a strong association between the percentages of spermatozoa with ZP-binding ability determined by our model and their IVF fertilization rates. The model outperformed the conventional semen analysis in predicting optimal/suboptimal IVF outcomes. This method enables clinical assessment of sperm fertilization potential independent of the existing parameters and identification of patients at risk of encountering IVF fertilization failure caused by spermatozoa with defective ZP-binding ability.

## Introduction

Infertility is a significant global health concern affecting about 15% of heterosexual couples of reproductive age, with approximately 20–70% of these cases attributed to male-factor infertility (Agarwal *et al.*, 2015). ART is the most effective treatment of infertility. *In vitro* fertilization (IVF) is the preferred method of insemination for men with normal semen parameters according to the World Health Organization (WHO) criteria (WHO, 2021). In IVF, motile spermatozoa need to bind onto the zona pellucida (ZP), the outer coat of an oocyte, to initiate fertilization. Alternatively, intracytoplasmic sperm injection (ICSI) is offered to couples with severe male factor infertility or a previous history of poor IVF fertilization rate to improve fertilization success rate (Payne *et al.*, 1994; Silber *et al.*, 1994; Leung *et al.*, 2022). ICSI is an invasive procedure that involves injecting a viable, microscopically normal-looking spermatozoon directly into an oocyte by micromanipulation. This approach might be associated with an increased risk of transferring paternal defects via the injected spermatozoa to the resulting child (Alukal and Lamb, 2008, Sciorio and Esteves, 2022), mainly due to circumvention of natural sperm selection barriers and the absence of a standard method for identifying spermatozoa without functional defects. Consequently, ICSI is not routinely recommended as the primary insemination method unless semen analysis indicates abnormalities in normal morphology (≤4%), concentration (≤15 M/ml), or motility (progressive motility+non-progressive motility) (≤40%) (Cooper *et al.*, 2010; WHO, 2021), with modifications subjected to in-house analysis among individual centres.

Sperm morphology is a key parameter assessed in semen analysis for investigating male infertility. The 5th percentile for normal sperm morphology determined by the strict criteria is ≥4% (WHO, 2021). However, the manual assessment of sperm morphology is highly labour-intensive, subjective, and operator dependent. Its predictive power has been challenged by studies showing a lack of association between sperm morphology and fertilization outcome following assisted reproduction (Shabtaie *et al.*, 2016; Gatimel *et al.*, 2017; Kohn *et al.*, 2018a). A meta-analysis involving 41,018 intrauterine insemination cycles concluded that the clinical pregnancy rates were similar between men with sperm morphology >4% and <4% (Kohn *et al.*, 2018b). In another study, the pregnancy rates following IVF or ICSI were comparable in spermatozoa with normal morphology >4% and ≤4% (Hotaling *et al.*, 2011). Despite its inherent limitations, conventional semen analysis results remain a crucial factor in selecting the most appropriate insemination method for assisted reproduction (O’Flynn, 2014). Approximately 10% of all couples (Bosch *et al.*, 2020) and 25% of couples with unexplained infertility (Johnson *et al.*, 2013) encounter fertilization failure following conventional IVF. Early detection of sperm samples with inferior fertilization potential can help determine the most suitable insemination method for the patients, thus preventing unexpected fertilization failure in ART.

Deep learning is an advanced approach in machine learning that utilizes artificial neural networks. Specifically, convolutional neural networks (CNNs) are a specialized class of deep-learning models that excel at identifying patterns and features for a wide range of medical applications, such as disease diagnostics by medical image classification (Iqbal *et al.*, 2020; Yu *et al.*, 2021) and object recognition aiding in drug delivery (Girshick *et al.*, 2016). In the past decade, deep learning has emerged as a popular approach for sperm morphology evaluation. Previous studies have reported on their initial success in classifying the morphological abnormalities of spermatozoa with good sensitivity and specificity during training (Thirumalaraju *et al.*, 2018, 2019; Riordon *et al.*, 2019; Iqbal *et al.*, 2020; Cherouveim *et al.*, 2023; Aktas *et al.*, 2024; Shahali *et al.*, 2024). However, all these models were trained with datasets annotated by the subjective judgement of andrology technicians according to the WHO strict criteria, which have limited predictive power on ART outcomes. Moreover, none of them have been clinically validated on their correlation between identified spermatozoa and fertilization outcome. Further studies are required to demonstrate the predictability of these algorithms in clinical IVF settings.

Spermatozoa–ZP binding is the first step in fertilization. ZP-binding ability is a quantitative, objective sperm quality metric related to genomic integrity (Leung *et al.*, 2023) as well as fertilization and pregnancy outcomes following conventional IVF (Liu and Baker, 2000; Sifer *et al.*, 2005), which makes it an ideal candidate for the application of deep-learning algorithms to improve the accuracy of sperm quality evaluations in clinical setting. Therefore, the objective of this study was to develop a unique deep-learning algorithm, which is markedly different from the conventional WHO criteria, to identify human spermatozoa with ZP-binding capabilities through image analysis of sperm morphology.

## Materials and methods

### Spermatozoa and oocyte collection

This study was approved by the Institutional Review Board of the University of Hong Kong/Hospital Authority Hong Kong West Cluster (UW19-828). Spare semen samples were collected from men undergoing premarital check-ups at the Family Planning Association of Hong Kong, with informed written consent. Normospermic samples were selected according to the WHO criteria, sixth edition (WHO, 2021): total volume ≥1.5 ml, total motility ≥40%, progressive motility ≥32%, total sperm count ≥39 × 10^6^ per ejaculate, concentration ≥15 × 10^6^/ml, viability ≥58%, and morphology ≥4%. The direct swim-up method and density gradient centrifugation (DGC) were used to isolate motile and viable spermatozoa from the seminal plasma as described previously (Leung *et al.*, 2023). Earles balanced salt solution (EBSS) (Fisher Scientific, Waltham, MA, USA) supplemented with 3% bovine serum albumin (EBSS/3%BSA) (Fisher Scientific) was used to induce capacitation in spermatozoa for subsequent coincubations (Chiu *et al.*, 2010).

Immature oocytes at germinal vesicle/metaphase I stage or mature metaphase II oocytes were donated from women attending the IVF clinic at Queen Mary Hospital, Hong Kong. Morphologically normal oocytes were collected and stored in high-salt oocyte storage buffer containing 1.5M MgCl_2_ (Sigma-Aldrich, Saint Louis, MO, USA), 0.1% polyvinyl pyrrolidone (Sigma-Aldrich), and 40 mM HEPES (Sigma-Aldrich), pH 7.2 at 4°C. They were used within a month upon collection. Morphologically abnormal oocytes were discarded.

### Laboratory samples of ZP-bound human spermatozoa for deep-learning model training

Acrosome-intact, ZP-bound spermatozoa were collected by our modified spermatozoa–ZP coincubation assay as described (Leung *et al.*, 2023) (Supplementary Fig. S1). Our previous report indicated that the ZP-bound spermatozoa recovered within 30 min remained acrosome-intact when compared to those recovered after a longer incubation period up to 2 h (Leung *et al.*, 2023). In brief, four human oocytes were co-incubated in a 30-µl droplet of EBSS/3% BSA containing 2 × 10^6^ spermatozoa covered with mineral oil at 37°C in 5% CO_2_ for 30 min. After incubation, the oocytes were successively washed in three droplets of EBSS/no BSA to dislodge loosely bound spermatozoa. The ZP-bound spermatozoa were then removed from the surface of the oocytes by vigorous aspiration using a fine-bored glass pipette in a confined area containing 10 µl of EBSS/no BSA on a sterile glass slide.

### Clinical samples of ZP-unbound human spermatozoa for deep-learning model training

ZP-unbound spermatozoa were collected from normozoospermic samples with defective ZP-binding ability, as evidenced by complete fertilization failure following conventional IVF and the absence of ZP-bound spermatozoa on the inseminated oocytes.

### Investigating the relationship between sperm ZP-binding ability and IVF fertilization rates using a deep-learning model

Male factor and unexplained infertility patients were categorized into three groups according to their IVF fertilization rates ranging, namely low: 0–40% (Donnelly *et al.*, 1998; Tian *et al.*, 2022), intermediate: 41–70%, and high: 71–100% (Liu and Baker, 2000; Rosen *et al.*, 2010). Their sperm samples were collected to examine the correlation between the ZP-binding ability of spermatozoa and IVF fertilization rates using the deep-learning model, and to determine a clinical threshold that can distinguish normal from defective ZP-binding ability of the processed sperm samples.

### Comparing the predictive value of fertilization rates in IVF between our deep-learning model and conventional semen analysis

Patients with male factor or unexplained infertility, who had either high or low fertilization rates in IVF, were selected to assess the clinical significance of the predicted percentages of spermatozoa with ZP-binding ability in comparison to their conventional semen analysis results.

### Diff-Quik staining of spermatozoa

All collected spermatozoa were smeared onto sterile glass slides and left to air-dry. The air-dried spermatozoa were then fixed in a methanol-based solution (RAL Diagnostics, Martillac, France) for 15 s. Subsequently, they were stained with a buffered Eosin Y solution (RAL Diagnostics) for 10 s, followed by staining with a buffered thiazine dye solution (RAL Diagnostics) for another 10 s. The excess solution was dripped off the slides between steps. Diff-Quik stained sperm images were captured under a light microscope (Zeiss, Gottingen, Germany) at a magnification of 1000× with oil immersion.

### Deep-learning model development

**Figure 1.**
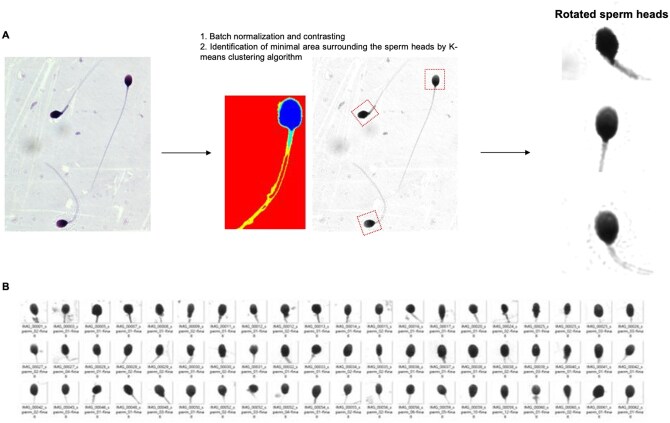
**Image processing and extraction of individual sperm heads from Diff-Quik stained images**. (**A**) An individual sperm head was identified by the standard K-means clustering algorithm in Diff-Quik stained images and cropped to a pixel size of 128×128. (**B**) Representative images of processed sperm heads.

An advanced CNN architecture named VGG13 (Simonyan and Zisserman, 2014), pretrained on ImageNet 1000 predefined classes, was further fine-tuned with our newly established database, which contained a total of 1083 Diff-Quik-stained images of ZP-bound and unbound spermatozoa. Colour-based segmentation using the K-means clustering algorithm was applied to extract individual sperm heads (Fig. 1A), which were cropped to a pixel size of 128 × 128, converted to greyscale, and aligned before use (Fig. 1B). Images of poor-quality spermatozoa, including those with debris and/or a dirty background, were manually removed from the dataset (Supplementary Fig. S2) to prevent the model from being misled and identifying irrelevant information during training. Clinical conditions might result in subtle microenvironmental differences, which were undetectable by naked eye but could greatly confuse the deep-learning classifier, leading to misinterpretation of datasets during training and validation. To address this issue, the Cycle Generative Adversarial Network (CycleGAN) (Zhu *et al.*, 2017) was employed to translate the distinctive features of laboratory samples into clinical samples and to adjust microenvironment conditions such as reducing background noise and debris, to ensure consistent image quality (Supplementary Fig. S3). The CycleGAN model was adapted from the original implementation by Zhu *et al.* (2017) and optimized using the Adam optimizer with a learning rate of 0.0002 and a momentum parameter (β1) of 0.5. The model was trained over 200 epochs, with the first 100 epochs at a constant learning rate, followed by a linear decay over the next 100 epochs. Least-squares GAN was used for the GAN loss objective, with a linear learning rate decay applied every 50 iterations. The training dataset comprised 1083 images of ZP-bound and unbound spermatozoa, randomly split into a 75% training set and a 25% validation set, with an additional 220 images reserved for testing.

Our VGG13 model, implemented in PyTorch, was adapted from the transfer learning tutorial on PyTorch by Chilamkurthy (2017). The model consisted of 10 convolutional layers with two components, namely a feature extractor and a classifier, which were linked by an average pooling layer. To finetune the classifier for the specific downstream task of sperm classification while leveraging the capability of the pretrained VGG13 model, the last layer of the network was modified while the previous layers were frozen with unchanged weights. The classifier was composed of three linear layers, where the first two were followed by a rectified linear unit (ReLU) layer and a dropout layer in a sequential manner. In the last fully connected linear layer, the number of output features was modified from 1000 to 2 for binary classification. Forward-propagation was performed to process the input data through successive hidden layers with 3 × 3 convolution filters. Additional data argumentations, including horizontal flipping and random resizing, were applied to further enhance the effective size and qualities of the training datasets. Stochastic gradient descent (SGD) was simultaneously performed to adjust the weights on individual nodes to minimize error rates. Cross-entropy loss was used to measure the difference between predicted probabilities and true class labels. The model was trained over 50 epochs, with a batch size of 4 and a learning rate of 0.001. The model was developed on Google Colab (Googleplex, Mountain View, CA, USA) using NVIDIA Tesla P100 or NVIDIA Tesla V100. Custom code was specific to our computing equipment used for image processing and binary classification.

### Classification performance

Confusion matrix was used to evaluate the model’s performance for binary classification by comparing the predicted labels to the actual labels, expressed in terms of accuracy, specificity, sensitivity, precision, and recall. A 5-fold cross-validation using K-fold on our training set was used to examine the training performance by dividing the training dataset into five equal parts. In each training, 1-fold was used as the test dataset and the rest was used as the training set. By repeating this process five times, the model was trained and tested on five individual, randomized datasets to examine its learning variance. Discriminative power was examined by the area under the receiver-operating characteristic (ROC) curve (AUC), with relevant pixel importance identified through saliency mapping. For clinical validation, logistic ROC regression analysis was used to estimate AUC and *P*-values for distinguishing high and low fertilization groups in clinical samples, with the optimal threshold identified by Youden’s index (sensitivity + specificity − 1).

### Data analysis

Clinical data were analysed using GraphPad Prism 9.1.0 (GraphPad Software, San Diego, CA, USA). Results are presented as mean ± SEM. A 2-tailed unpaired *t*-test was conducted to assess differences in: (i) pixel importance localized to spermatozoa relative to the entire image between unconverted and GAN-converted images, and (ii) fertilization rates between groups with 0–40% and 71–100%. In the cases where the data failed the normality test, Mann–Whitney (non-parametric) test was used to perform statistical analyses. A probability value of <0.05 was considered statistically significant.

## Results

### Development of a VGG13 model for binary classification of ZP-bound and unbound spermatozoa

**Figure 2.**
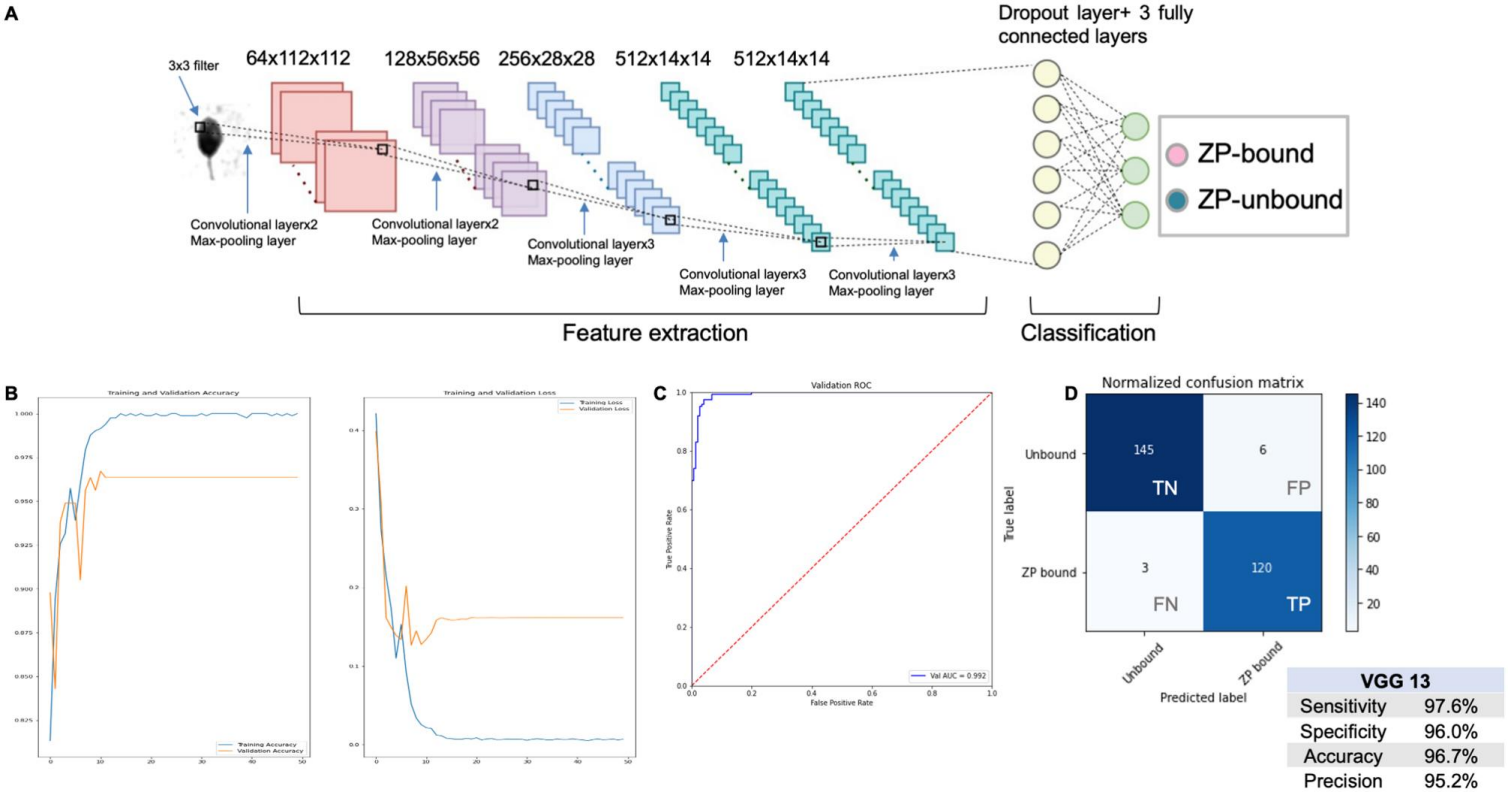
**Graphic illustration of our VGG13-based method to evaluate the zona pellucida (ZP)-binding ability of human spermatozoa**. (**A**) The images were inputted into the VGG13 model consisting of successive hidden layers for binary classification. The model comprised a feature extractor and a classifier, which were connected by an average pooling layer. (**B**) Accuracy curve: to evaluate the overall reliability of the model (Left). Cost curve: to measure the error rates of classifications (Right). (**C**) Receiver-operating characteristic curve: to examine the discriminative power of the model as reflected by the AUC value. (**D**) Confusion matrix: to measure the classification performance of the model for binary classification. TN, true negative; FN, false negative; FP, false positive; TP, true positive.

**Figure 3.**
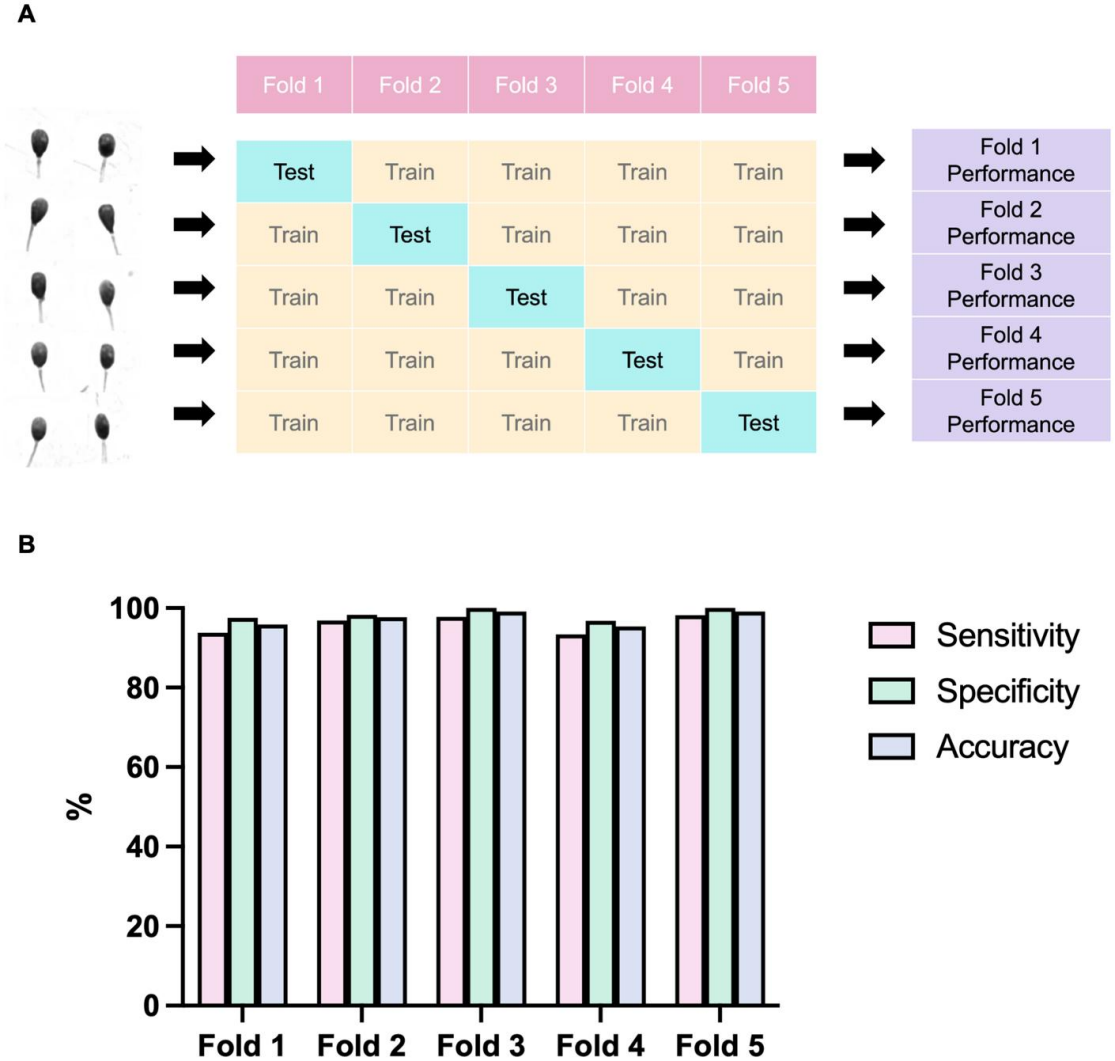
**K-fold cross-validation of the fine-tuned VGG13 model**. (**A**) Graphic illustration of the 5-fold data splitting and cross-validation. In each run, the entire dataset was randomly split into five parts, with one part used as a test dataset and the remaining four parts used as a training dataset to evaluate the model’s reliability and accuracy. (**B**) Classification performance evaluated across five folds of datasets with low learning variance. All combinations of datasets demonstrated exceptional performance with high rates of sensitivity, specificity, and accuracy.

We fine-tuned a VGG13 model using our database of ZP-bound spermatozoa, collected through our established experimental protocol, and clinical samples of unbound spermatozoa with defective ZP-binding ability (n = 1083). The newly fine-tuned VGG13 model, constructed as shown in Fig. 2A, demonstrated success in classifying the ZP-bound and unbound spermatozoa with a high accuracy and a low cost (a measure of the total error rates) during training (Fig. 2B) over 50 epochs. The model was trained on the datasets using a batch size of 4 with a learning rate ranging from 0.0001 to 0.1 (Supplementary Fig. S4). The results indicated that a high learning rate negatively impacted the weight adjustment with respect to a loss gradient during training. Therefore, 0.001 was selected as the optimal learning rate for our model, as reflected by the high accuracy (Supplementary Fig. S4). The fine-tuned model discriminated the ZP-bound spermatozoa from the unbound ones based on their morphological features, with an AUC of the ROC curve of 0.992 (Fig. 2C). It exhibited good classification performance with high sensitivity (97.6%), specificity (96.0%), accuracy (96.7%), and precision (95.2%), as determined by the confusion matrix (Fig. 2D). Moreover, the model demonstrated low learning variance (Fig. 3) across five randomly split datasets for training and testing, achieving an average accuracy of 97.4%, sensitivity of 96.0%, and specificity of 98.5%. Overall, the results indicated that the model was able to differentiate the morphological features between ZP-bound and unbound spermatozoa, without displaying any indications of overfitting to our representative datasets.

A comparison was also conducted between the classification performance of the VGG13 and ResNet34 models. ResNet34, which consists of 33 convolutional layers with skip connections and an additional fully connected layer, facilitates effective training for image classification (He *et al.*, 2016). When fine-tuned, ResNet34 achieved high classification metrics similar to our fine-tuned VGG13 model: sensitivity (98.4% vs 97.6%), specificity (100% vs 96.0%), accuracy (99.3% vs 96.7%), and precision (100% vs 95.2%) (Supplementary Fig. S5). However, it exhibited limited discriminatory power between ZP-bound and unbound spermatozoa in the test dataset when compared to the VGG13 model, likely due to overfitting. Notably, 25.5% of unbound spermatozoa were misclassified as ZP-bound using the ResNet34 model, compared to only 1.0% by the VGG13 model (Supplementary Fig. S6). Consequently, VGG13 was chosen as the primary model for the binary classification of ZP-bound and unbound spermatozoa in our study.

### Effects of transfer learning on deep-learning model classification performance

Transfer learning was employed to fine-tune the VGG13 model on our dataset of ZP-bound and unbound spermatozoa. This technique allowed the model to retain valuable information from the ImageNet dataset’s 1000 predefined classes for feature extraction and adapt to the new task by adjusting its parameters based on our dataset. The efficacy of transfer learning for binary classification of spermatozoa was validated by comparing the performance of a basic CNN model trained from scratch with our fine-tuned VGG13 model. The CNN model, consisting of two convolutional layers and one max pooling layer (Supplementary Fig. S7A), was trained on our datasets with a batch size of 6 over 50 epochs and a learning rate of 0.01 (Supplementary Fig. S7B). Although this model could differentiate the sperm subpopulations to some extent (Supplementary Fig. S7C), its overall classification performance (Supplementary Fig. S7D; Sensitivity: 87.8%, Specificity: 76.2%, Accuracy: 81.3%, and Precision: 74.3%) was inferior to the fine-tuned VGG13 model. The CNN model incorrectly classified 68.4% of the unbound spermatozoa images in the test dataset as ZP-bound spermatozoa, indicating poor generalization (Supplementary Fig. S8A). In contrast, the fine-tuned VGG13 model successfully discriminated between the two classes of spermatozoa for ∼99% of the images, demonstrating excellent generalization ability (Supplementary Fig. S8A and B). These results indicate that the fine-tuned VGG13 model, trained using transfer learning, is the optimal approach for distinguishing between ZP-bound and unbound spermatozoa.

### Interpretability analysis of the fine-tuned VGG13 model

**Figure 4.**
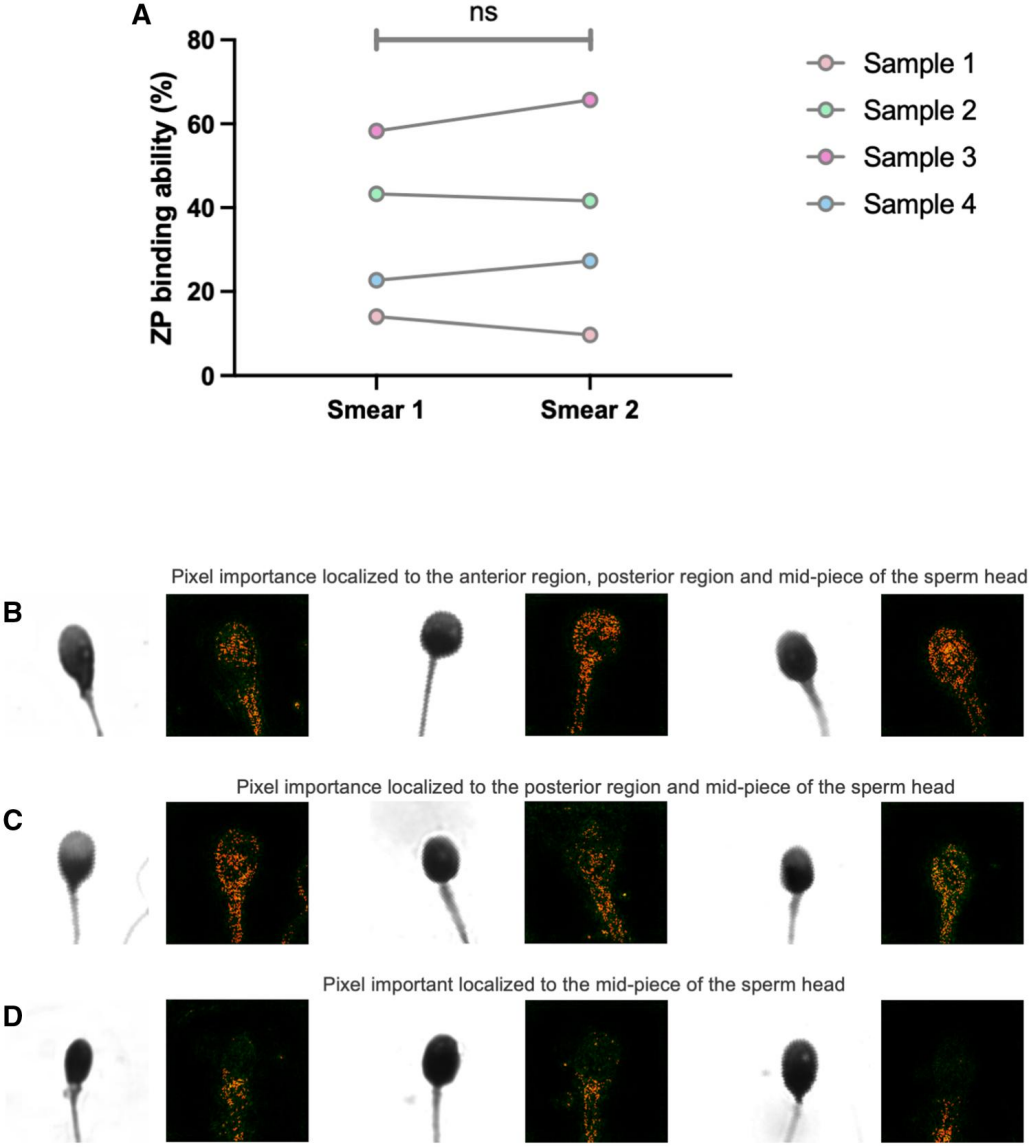
**Interpretability analysis of the fine-tuned VGG13 model**. (**A**) Evaluation of prediction reproducibility. Four sets of samples were collected and processed using our established protocols as described. Two individual slides of each sample were inputted into the model to determine the predicted percentage of spermatozoa with zona pellucida (ZP)-binding ability. The low variation in the predicted values between slides indicated that the model was able to generate comparable output for different sets of images of the same sample. (**B–D**) Saliency maps for representative images of human spermatozoa from our training datasets. The saliency map overlay highlighted the relevant features that contributed to the prediction output of the model, as shown by the pixel importance in orange. Representative images displaying localization pattern of pixel importance on sperm images. (B) Pixel importance clustering over the anterior region, posterior region, and midpiece of the sperm heads. (C) Pixel importance clustering over the posterior region and midpiece of the sperm heads. (D) Pixel importance clustering over the midpiece of the sperm heads.

The observed variations across two individual sets of images of samples were low, demonstrating that the model had a high prediction reproducibility (Fig. 4A). Saliency maps (Fig. 4B–D) indicated that the model focused on regions corresponding to the sperm head and mid-pieces in all images, with minimal attentions to the background noise and debris. Analysis of heat maps generated using our training datasets revealed that the localization pattern of the pixel importance varied significantly (Fig. 4B–D) between individual spermatozoa but primarily clustered to the anterior regions of the sperm head when compared to the posterior region and mid-piece, in both ZP-bound and unbound spermatozoa. In most cases, the model recognized the distinctive region known to be involved in ZP-binding interaction, shedding light on the decision-making process used within the black-box approach.

### Interpretability analysis of the CycleGAN model for image conversion

We used saliency maps to qualitatively analyse pixel importance, focusing on spermatozoa in both unconverted and CycleGAN-converted images. A VGG13 model was fine-tuned on a dataset of ZP-bound and unbound spermatozoa, achieving good classification performance as shown in the confusion matrix (Supplementary Fig. S9). Saliency map analysis (Supplementary Fig. S10A–C) revealed that in unconverted images, only about 51.4% of pixel importance was localized to the spermatozoa, while 48.6% was distributed over irrelevant components like background noise and debris. In contrast, CycleGAN-converted images showed ∼91.6% of pixel importance clustered over the spermatozoa (Supplementary Fig. S10D).

### Clinical validation of the fine-tuned VGG13 model for predicting fertilization outcomes following IVF

**Figure 5.**
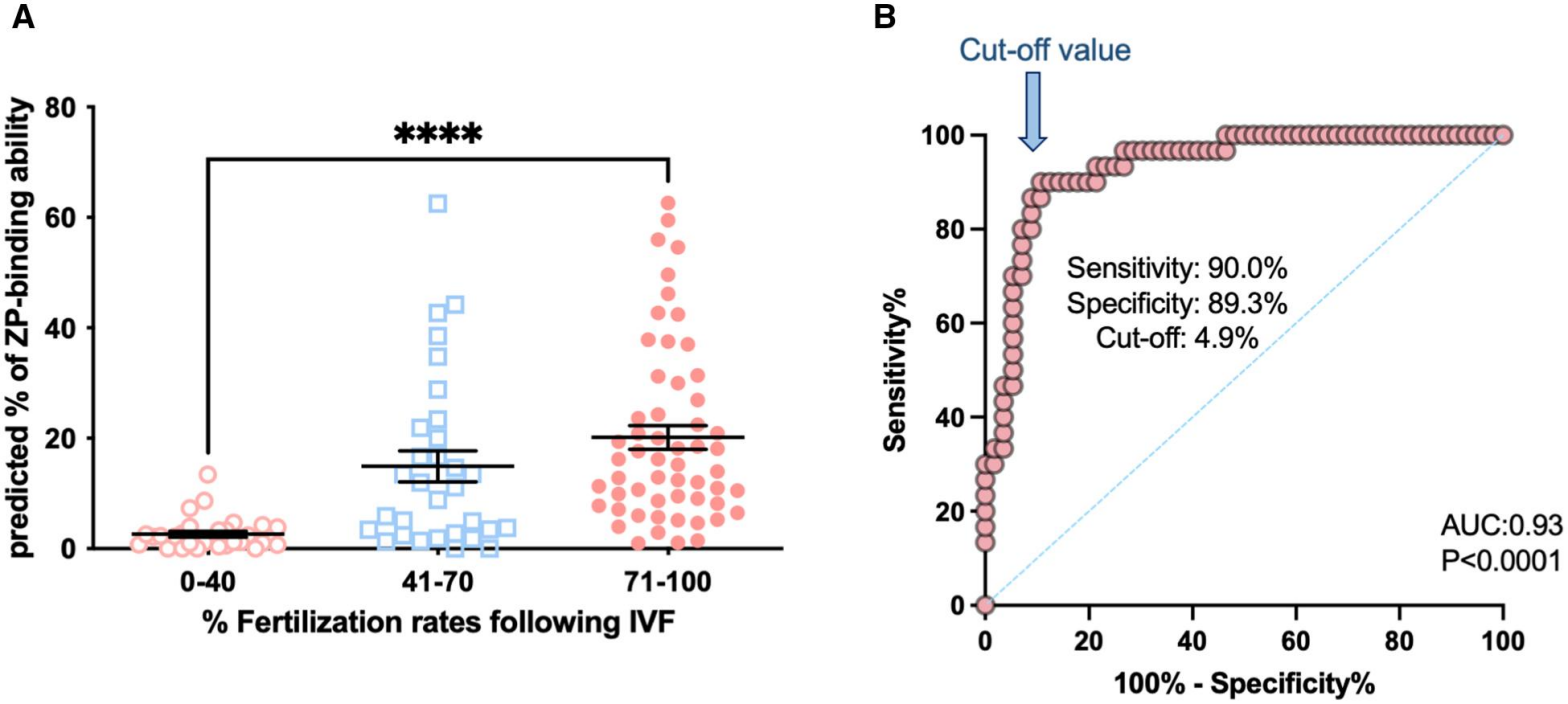
**Examination of the predictive power of the fine-tuned VGG13 model for fertilization outcome following IVF**. Diff-Quik stained images of three groups of men (n = 117; *P* < 0.001), with fertilization rates of 0–40%, 41–70%, and 71–100%, were collected as described. (**A**) Association between the predicted percentage of spermatozoa with zona pellucida (ZP)-binding ability and fertilization rates following IVF. All data are represented as mean±SEM. (**B**) Identification of the optimal threshold for clinical prediction. Logistic receiver-operating characteristic regression was established using our predicted values of the high and low fertilization groups (n = 86). **** indicates statistical significance between the high and low fertilization rate groups (*P* < 0.0001).

Three groups of men (n = 117) with fertilization rates of 0–40% (Low), 41–70% (Intermediate), and 71–100% (High) were recruited from our assisted reproduction program to examine the relationship between the ZP-binding ability assessed by deep learning and their fertilization rates following IVF (Fig. 5A). The results demonstrated that the average percentage of spermatozoa with ZP-binding ability varied distinctively among the three groups (Low: 2.6 ± 0.5%; Intermediate: 14.9 ± 2.8%; High: 20.2 ± 2.2%). The high fertilization rate group had a significantly higher predicted percentage of ZP-bound spermatozoa than the low fertilization rate group (Fig. 5A; *P* < 0.05), consistent with a previous report on differences in the number of motile spermatozoa with ZP-binding ability between fertile and infertile men (Liu and Baker, 2000).

The fine-tuned VGG13 model exhibited robust generalization to unseen data, achieving an AUC of 0.93 in distinguishing between high and low fertilization rate groups (*P* < 0.0001, n = 86; Fig. 5B). The Youden’s index was used to identify the optimal threshold value on the ROC curve. A cut-off value of 4.9% with a specificity of 89.3% and a sensitivity of 90.0% was established as the clinical threshold to distinguish between normal and defective ZP-binding ability of the DGC-processed sperm samples.

### Clinical significance of the fine-tuned VGG13 model in comparison with the conventional semen analysis

The clinical implications of the predicted percentages of spermatozoa with ZP-binding ability per sample (generated by the fine-tuned VGG13 model) were further validated by comparing its predictive power to IVF fertilization outcome with the conventional semen analysis results among additional 30 patients with known fertilization rates (Table 1). All the patients (n = 30) presented normal semen analysis results that satisfied our laboratory criteria for conventional IVF on the day of insemination. In the groups of patients with high fertilization rates (71–100%), notably, our model determined patients 2, 3, 10, and 12 as having normal ZP-binding ability above the clinical threshold of 4.9%, despite their normal morphology results below the WHO reference value. Furthermore, our model successfully identified patients 17, 19–21, 23, 24, 26, and 30 who were likely to experienced suboptimal fertilization rates (0–40%) despite having normal semen parameters. In these cases, the predicted values contradicted the semen analysis results but strongly aligned with the IVF fertilization rates. These findings highlight the prognostic value of our model in evaluating sperm fertilization potential. Additionally, the data suggest that ZP-binding ability could serve as an additional measure of sperm quality, complementing conventional semen parameters.

**Table 1.** **Comparison of clinical relevance between the conventional semen analysis and the fine-tuned VGG13 model**.

	Raw semen samples				Semen analysis of density gradient centrifugation-processed spermatozoa			Predicted ZP-binding ability % as determined by our invention	Recommended for IVF?		IVF fertilization rates%
	Progressive motility%	Non-progressive motility %	Sperm concentration (μM)	Morphology %	Progressive motility%	Non-progressive motility %	Sperm concentration (μM)		Semen analysis result	ZP-binding analysis result (clinical threshold ≥4.9)	
Patient 1	53	14	31.7	4	76	18	8.2	52.8	Yes	Yes	100 (High)
Patient 2	45	16	45.9	**1**	77	15	4.5	**25**	Yes	Yes	100 (High)
Patient 3	25	4	140.6	**2**	81	13	18.5	**41.2**	Yes	Yes	100 (High)
Patient 4	36	16	322.5	4	55	17	18.3	15.3	Yes	Yes	100 (High)
Patient 5	55	15	107.9	4	74	19	53.4	58.4	Yes	Yes	100 (High)
Patient 6	55	5	137.8	6	81	14	26.1	33.9	Yes	Yes	83.3 (High)
Patient 7	41	11	34.1	5	88	2	28.4	38.3	Yes	Yes	88.9 (High)
Patient 8	44	11	37.7	5	72	10	10.1	20.5	Yes	Yes	100 (High)
Patient 9	46	9	45.2	4	70	14	27.1	20	Yes	Yes	75 (High)
Patient 10	45	6	265	**2**	73	7	21.2	**5**	Yes	Yes	100 (High)
Patient 11	36	10	41.7	5	65	31	4.9	6.3	Yes	Yes	100 (High)
Patient 12	37	15	31.9	**2**	56	9	3.1	**34.6**	Yes	Yes	75 (High)
Patient 13	38	5	34.6	6	80	10	24.4	7.7	Yes	Yes	100 (High)
Patient 14	43	8	26.4	4	78	6	32.3	12.6	Yes	Yes	100 (High)
Patient 15	52	7	52.6	7	74	4	28.4	17	Yes	Yes	81.8 (High)
Patient 16	61	8	33.1	4	76	6	11.5	31.9	Yes	Yes	77.8 (High)
Patient 17	51	9	46.5	**4**	88	9	5.7	**1.6**	Yes	No	9.1 (Low)
Patient 18	25	3	121	2	72	12	15.5	2.9	Yes	No	33 (Low)
Patient 19	47	4	59.9	**4**	86	11	14.7	**4**	Yes	No	0 (Low)
Patient 20	45	5	124.4	**4**	67	22	14.2	**3.6**	Yes	No	30 (Low)
Patient 21	61	10	71.1	**5**	84	12	29.6	**0.9**	Yes	No	24 (Low)
Patient 22	44	12	134.1	3	48	14	27.5	4.4	Yes	No	30 (Low)
Patient 23	64	9	134.7	**6**	79	35	26.6	**0**	Yes	No	0 (Low)
Patient 24	47	4	27.7	**4**	76	6	8.6	**3.6**	Yes	No	33.3 (Low)
Patient 25	22	14	71	2	69	9	6.5	2.1	Yes	No	0 (Low)
Patient 26	42	15	8.3	**4**	66	27	9.1	**1.6**	Yes	No	0 (Low)
Patient 27	45	14	37.1	3	65	10	4.4	3.9	Yes	No	33.3 (Low)
Patient 28	22	3	23.8	4	83	8	3.6	2.9	Yes	No	25 (Low)
Patient 29	42	3	26.2	3	81	17	5.5	3.2	Yes	No	0 (Low)
Patient 30	52	7	52.6	**7**	72	21	12.7	**1.7**	Yes	No	33.3 (Low)

Clinical data of 30 patients undergoing conventional IVF were collected from our IVF clinic to further validate the clinical significance of our model. All patients recruited in this study had normal semen analysis results as determined by the embryologist and received conventional insemination as the primary fertilization method. The residual sperm samples of these patients were further examined to evaluate the predicted percentages of spermatozoa with zona pellucida (ZP)-binding ability (clinical threshold: 4.9%) using our model as described. In the high fertilization rate group, patients #2, 3, 10, and 12 (highlighted in bold type) were found with normal ZP-binding ability above the clinical threshold, despite having normal morphology below the reference value. In the low fertilization rate group (highlighted in bold type), patients #17, 19–21, 23, 24, 26, and 30 were identified as individuals at risk of encountering IVF fertilization failure due to defective ZP-binding ability, despite having a percentage with normal morphology above the reference value.

## Discussion

Our findings showed that deep learning can be used to predict the fertilization potential of spermatozoa based on their morphological characteristics associated with ZP-binding ability. The fine-tuned model is the first algorithm demonstrated to have excellent generalization ability and high discriminative power in classifying sperm subpopulations with fertilization potential, as shown by the strong correlation between the predicted ZP-binding ability and IVF fertilization rates. An automated system integrating with our clinical threshold can allow real-time assessment of the ZP-binding ability of clinical samples, complementing the conventional semen analysis results for predicting fertilization outcome following IVF.

Conventional semen analysis is a standard assessment of the fertility potential of men, examining surrogate parameters including sperm motility, concentration, and morphology. Although some studies found correlations between individual semen parameters and time-to-pregnancy (Bonde *et al.*, 1998; Zinaman *et al.*, 2000; Buck Louis *et al.*, 2014), a multivariate model that considered multiple parameters showed limited predictive power for fertilization outcome. This suggests that conventional semen analyses may not provide a comprehensive assessment of male fertility potential, as there is a compensatory effect between different semen parameters. The current methodology of sperm morphology assessment may explain the lack of its correlation to the fertilization outcome. First, manual evaluations of sperm morphology even by trained technicians exhibit high variation (Menkveld, 2013; Wang and Swerdloff, 2014). Second, morphological examinations of 200 spermatozoa, as recommended by WHO, may not truly represent the entire sample due to the large variation in morphological features among individual spermatozoa. While increasing the number of spermatozoa evaluated per sample can address this limitation, this practice is labour-intensive and costly in clinical settings. It is therefore imperative to develop a highly robust, automated method to evaluate the fertilization potential of spermatozoa before commencing IVF to improve the overall clinical management.

Despite major technological advancements, fertilization failure occurs in 5–10% of ART cycles using conventional insemination (Mahutte and Arici, 2003). A recent large retrospective cohort study concluded that the predictive power of semen parameters assessed in conventional semen analysis for total fertilization failure following conventional IVF was limited (Jiang *et al.*, 2023). ZP-binding ability of spermatozoa is an independent, surrogate matric of sperm quality closely related to high rates of acrosome reaction and DNA integrity (Liu and Baker, 2007; Liu *et al.*, 2007), and positively correlated with fertilization rates following IVF (Bastiaan *et al.*, 2003; Liu and Baker, 2003). The hemizona assay was developed to assess the ZP-binding ability of spermatozoa by incubating matching, equally bisected hemizona with samples from fertile men and patients for clinical diagnosis, and the assay can identify male-factor patients at risk of fertilization failure (Oehninger *et al.*, 2013). However, its clinical application is limited due to the complicated micromanipulation procedure in bisecting the ZP and the limited availability of human oocytes.

CNNs are structured as interconnected nodes in multiple layers, resembling the decision-making processes in the human brain. During training, CNNs assign weights to each feature based on its relevance to the final output (Alzubaidi *et al.*, 2021a; Chen *et al.*, 2021). Starting from the first layer, the model focuses on low-level features such as edges and corners, progressively extracting hierarchical visual information. By extracting high-level discriminative features in subsequent layers, the model increases the complexity of the identification process. A well-trained CNN can generalize its analysis to unseen data collected from the same condition as the training datasets. The ability of deep learning to generalize to unseen data is highly dependent on the quality of the training dataset. When manual handling is involved in the process, it is bound to influence the automaticity of the model in data extraction and its ability to reproduce results accurately when exposed to unseen data. Ideally, deep learning should be inherently capable of learning from the input data with minimal human intervention to enable automated extraction of hidden pattern in input data. In this study, we maximized the automated power of deep learning by allowing it to fine-tune on a novel dataset of spermatozoa with or without ZP-binding ability based on their morphological features. ZP-bound spermatozoa were collected using our established protocol under optimal conditions so that their ZP-binding ability was the sole factor contributing to the binding efficacy and so that they remained morphologically unchanged prior to acrosome reaction, thus preserving their structural integrity. On the other hand, unbound spermatozoa were pooled from clinical samples with complete fertilization failure following conventional IVF, with the absence of ZP-bound spermatozoa on the inseminated oocytes. ZP-binding receptor is a multimeric protein complex(es) collectively assembled during capacitation, as evidenced by the lack of a single gene mutation in spermatozoa can cause complete fertilization failure in human and mice (Chiu *et al.*, 2014; Leung *et al.*, 2023). Considering the rare incidence of multiple gene mutations in one individual, it is unlikely that this is the underlying mechanism leading to null ZP-binding across all participants in our study. Therefore, it is logically assumed that the unbound spermatozoa were representative of those with defective ability at a single-cell level. Moreover, fertile men ejaculate millions of spermatozoa, of which only ∼8–25% of them can bind to the ZP (Liu *et al.*, 2003; Leung *et al.*, 2023), indicating the presence of a high proportion of spermatozoa with defective ZP-binding ability produced in spermatogenesis. It is likely that the infertile, normospermic men recruited for our study produced spermatozoa with defective ZP-binding ability to a greater extent compared with the fertile individuals.

This approach enabled us to establish a new, previously unknown parameter relevant to sperm fertilization potential for a comprehensive profile of male fertility status. Individual sperm heads up to the neckpieces were extracted from the images by grouping images with a vector of pixel values similar to a reference image using K-means clustering algorithm. The clinical implications of the morphological features of the sperm tails were much more complex compared to those of the sperm heads, mainly due to the lack of standardized criteria for sperm tail morphology assessment and high variations among individual spermatozoa within a single sample. It has been suggested that the presence of sperm tail within the visual range led to biased, unreliable predictions of DNA quality in relation to sperm head morphology using deep learning (McCallum *et al.*, 2019).

CycleGAN was used to address the potential microenvironment disparity between laboratory samples of ZP-bound spermatozoa and clinical samples of unbound spermatozoa. This approach allowed for the translation of the unique features of ZP-bound spermatozoa into clinical samples, resulting in a more consistent dataset representative of sperm morphology. CycleGAN is a powerful type of GAN designed to transfer acquired knowledge between two different image domains for image-to-image translation (Welander *et al.*, 2018). CycleGAN consists of two generators and two discriminators to produce plausible and high-quality images by training in opposition to each other. To be more specific, the generators create converted images in one domain, which are evaluated by the discriminators for authenticity, mainly for the determination of how convincing the converted images appear when compared to the reference images from the target domain.

Our results demonstrated the high feasibility of image conversion using the cycle GAN model. However, the model was trained on a dataset captured under specific optimized settings, which may limit its ability to generalize to images acquired under different conditions. To improve the model’s generalization capabilities for future clinical applications, it is crucial to enhance the diversity and size of the training dataset. This can be achieved by exposing the model to a wide range of variations across clinical datasets, incorporating data augmentation (Takahashi *et al.*, 2020), and employing regularization techniques such as dropout, weight decay, and early stopping based on validation performance (Lee and Seok, 2020). These measures will ensure high generalization ability and minimize the risk of introducing artifacts or medically irrelevant features due to overfitting.

Training a deep-learning model from scratch with the ability of generalization to unseen data requires a substantial amount of training data and computational power. Transfer learning is commonly used to fine-tune the classifier of a pre-trained model for a new task by retraining the final layers of the model on a small dataset while retaining all the optimized weights acquired from a large, pre-existing dataset (Tajbakhsh *et al.*, 2016). This approach is particularly useful in medical image analysis (Byra *et al.*, 2018; Alzubaidi *et al.*, 2020, 2021b) to address the limitations of sample availability and manual annotations, which can significantly improve classification accuracy and training efficiency without overfitting. It has been used to fine-tune two pre-trained deep-learning models for sperm segmentation to automatically identify and label the sperm head, acrosome, and nucleus, with an average agreement of 95% with the manually segmented regions (Marín and Chang, 2021). Another study further explored the use of this technique to classify sperm morphology according to the WHO criteria, with an accuracy of 96.0% and a precision of 96.4% (Liu *et al.*, 2021).

Considering the limited availability of ZP-bound and unbound spermatozoa, the VGG13 model was used as a feature extractor to identify relevant and distinctive features of our input images, which were then used as training data to develop a new classifier for the specific task of binary classification with high specificity and sensitivity. A basic CNN model was also trained from scratch to further examine the advantages of transfer learning on the limited datasets. Although the CNN model demonstrated reasonable classification performance, it lacked the ability to effectively generalize to unseen clinical data that were derived from the same condition as the training set. One possible explanation is that the model failed to extract unique and distinctive features from the small datasets to establish specific patterns of representation due to the high morphological variations among individual spermatozoa within each class.

Deep-learning models do not generally provide comprehensive information regarding the decision-making process owing to their uninterpretable ‘black box’ nature. Saliency map is one method to visualize and conceptually interpret the decision-making process by revealing the pixels localized to the specific region(s) of an image that contribute to the classification process (Simonyan *et al.*, 2013). This technique has been used to validate the effectiveness of deep learning to identify the regions of interest, such as abnormalities and cell types in medical images (Bernal *et al.*, 2015; Poplin *et al.*, 2018; Ayhan *et al.*, 2022). Our model revealed that the pixels localized to the anterior region are a major morphological factor contributing to its learning and classification processes, consistent with the biological significance of the sperm acrosomal region for ZP-binding.

The average percentages of spermatozoa with ZP-binding ability varied significantly among the three fertilization groups, but there was a considerable overlap among some patients in the groups. This may be due to the high morphological variations among spermatozoa in a sample, possibly linked to developmental arrest during spermatogenesis. Since ZP-binding ability is measured collectively, the presence of immature spermatozoa can affect the overall predicted values. In addition, compensatory effects from other sperm parameters may explain why ZP-binding ability alone does not determine fertilization success. For instance, spermatozoa with hyperactivated motility may improve their chances of interacting with the ZP to initiate fertilization, even if the model predicts a low percentage of spermatozoa with ZP-binding ability.

Considering the high overall predictive power of ZP-binding ability for fertilization outcome following IVF in our study, the data generated by the model were used to determine the threshold to distinguish between normal and defective ZP-binding ability of spermatozoa. When compared with the conventional semen results alone, our model provided additional information on sperm fertilization potential, irrespective of the sperm parameters. Our model enables identification of men who are likely to fail in conventional IVF due to defective ZP-binding ability (≤4.9%) despite having normal semen parameters in semen analysis. Taken together, the ability of spermatozoa to bind to the ZP can serve as an independent indicator of their functional competency, enhancing conventional semen analysis in predicting IVF fertilization outcomes. Incorporating ZP-binding ability in initial assessments can be especially useful for identifying men whose spermatozoa appear normal by conventional parameters but have underlying functional defects. By providing patients with a more comprehensive profile of male fertility potential, our newly fine-tuned model offers personalized approach to fertility management and enables informed decision on the insemination method (conventional IVF vs ICSI) to be used in the first cycle, preventing them from psychological and financial suffering caused by fertilization failure in conventional IVF.

Currently, our model is only applicable to Diff-Quik stained images captured at high resolution while its use with other staining methods remains to be optimized. A deep-learning model has been established to detect morphologically abnormal spermatozoa from non-stained and relatively low-resolution images (Javadi and Mirroshandel, 2019). Our model is being optimized for non-stained sperm images in a comparable manner. Two deep learning-based models have been trained on stain-free spermatozoa of known DNA quality for predictions with brightfield images (McCallum *et al.*, 2019; Noy *et al.*, 2023). Nevertheless, our model will need to be tested on datasets of semen samples collected under different preparation conditions, such as variations in sample processing method and staining protocol, to ensure consistent classification accuracy. Our current model can serve as a foundation for developing an advanced platform that combines microfluidics and deep learning to simultaneously identify and separate high-quality viable sperm cells for ICSI. Microfluidics have been used to isolate specific sperm subpopulations based on their biological parameters and physiological properties (Nosrati *et al.*, 2017). Using a high-resolution, time-lapse imaging system, the integrated platform can examine and instantly select motile spermatozoa based on their morphological features through deep learning. Deep learning has been successfully employed to analyse video recordings of sperm motility (Hicks *et al.*, 2019; Valiuškaitė *et al.*, 2020), showcasing the potential of this technology for live sperm selection.

## Conclusion

Our newly fine-tuned model incorporating ZP-binding ability allows identification of spermatozoa by Diff-Quik stained image analysis using deep learning, which can achieve: (i) clinical assessment of sperm fertilization potential independent of those derived from the conventional semen analysis; and (ii) diagnostic identification of infertile patients with defective ZP-binding ability. In particular, the clinical threshold for ZP-binding ability can be used to identify men whose spermatozoa possess inferior fertilizing ability, which may lead to a high likelihood of fertilization failure in IVF. The development of a reliable and robust method for sperm evaluation enables informed decision-making between ICSI and conventional insemination, potentially leading to improved fertilization results. Ultimately, this technology may contribute to a better clinical management of infertile couples undergoing assisted reproduction treatments.

## Supplementary Material

hoaf024_Supplementary_Data

## Data Availability

The data underlying this article will be shared on reasonable request to the corresponding author.
