# Supplementary material for: Automatic identification of human spermatozoa with zona pellucida-binding capability using deep learning
Source: Hum Reprod Open. 2025 May 10;2025(3):hoaf024. doi: 10.1093/hropen/hoaf024 (PMC12145210; doi:10.1093/hropen/hoaf024)
Supplement: hoaf024_Supplementary_Data [file hoaf024_supplementary_data.pdf]

Supplementary Data

Automatic identification of human spermatozoa with zona pellucida-binding capability using deep learning

Erica T.Y. Leung[1], Xianghan Mei[2], Brayden K. M. Lee[1], Kevin K.W. Lam[1,3], Cheuk-Lun Lee[4], Raymond H.W. Li[1,3], Ernest H.Y. Ng[1,3], William S.B. Yeung[1,3,5*], Lequan Yu[2*], Philip C.N. Chiu[1,5*]

1 Department of Obstetrics and Gynecology, School of Clinical Medicine, Li Ka Shing Faculty of Medicine, The University of Hong Kong, Hong Kong

2 Department of Statistics and Actuarial Science, The University of Hong Kong, Hong Kong

3 Department of Obstetrics and Gynecology, Queen Mary Hospital, Pok Fu Lam Road, Hong Kong

4 Department of Health Technology and Informatics, The Hong Kong Polytechnic University, Hong Kong

5 Hong Kong-Shenzhen Key Laboratory of Fertility Regulation, The University of Hong Kong-Shenzhen Hospital, Shenzhen, China

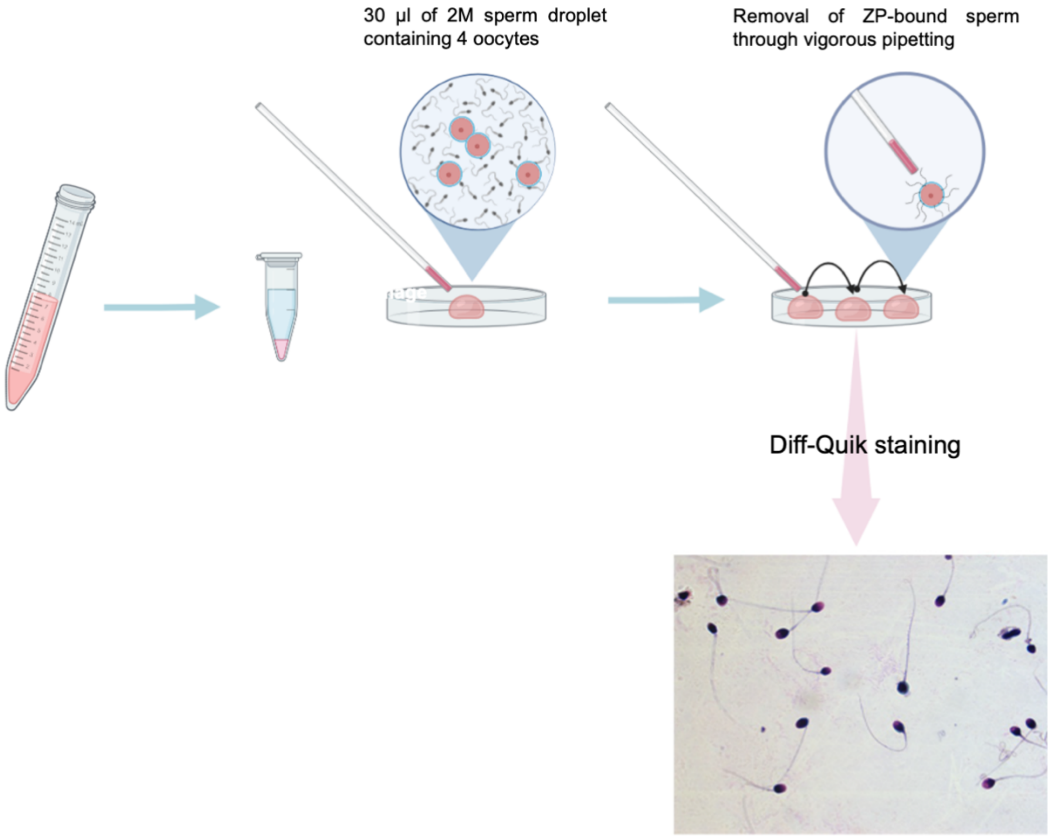

Supplementary Figure S1. Graphic illustration of spermatozoa-ZP coincubation to collect ZP-bound spermatozoa. A group of 4 oocytes was co-incubated in a 30 µL droplet of EBSS/3% BSA containing 2×10^6 spermatozoa covered with mineral oil at 37°C in 5% CO_2 for 30 min. After incubation, the oocytes were successively washed in 3 droplets of EBSS/no BSA to dislodge loosely bound spermatozoa. The ZP-bound spermatozoa were then removed from the surface of the oocytes by vigorous aspiration using a fine-bored glass pipette in a confined area containing 10 µL of EBSS without BSA on a sterile glass slide. The collected ZP-bound spermatozoa were allowed to air-dry and subjected to Diff-Quik staining as described. The images were captured under a light microscope at a magnification of 1000x with oil immersion. ZP: zona pellucida; EBSS: Earles balanced salt solution; BSA: bovine serum albumin.

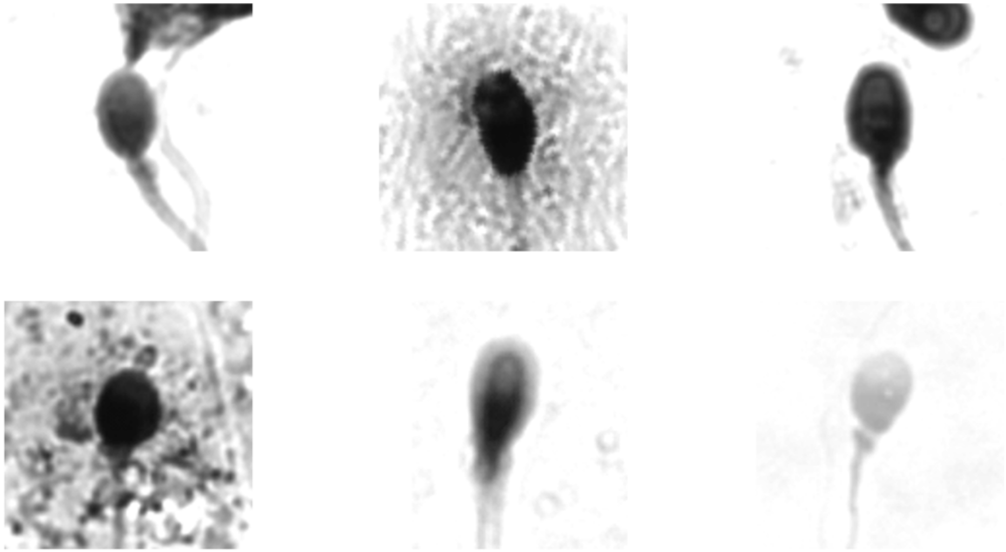

Supplementary Figure S2. Representative sperm images of poor-quality. The images of extracted sperm heads with undesirable features such as noisy background and blurry details were manually remove from the datasets to ensure comprehensive representation of sperm morphology, preventing the model from being misled and identifying irrelevant information during training.

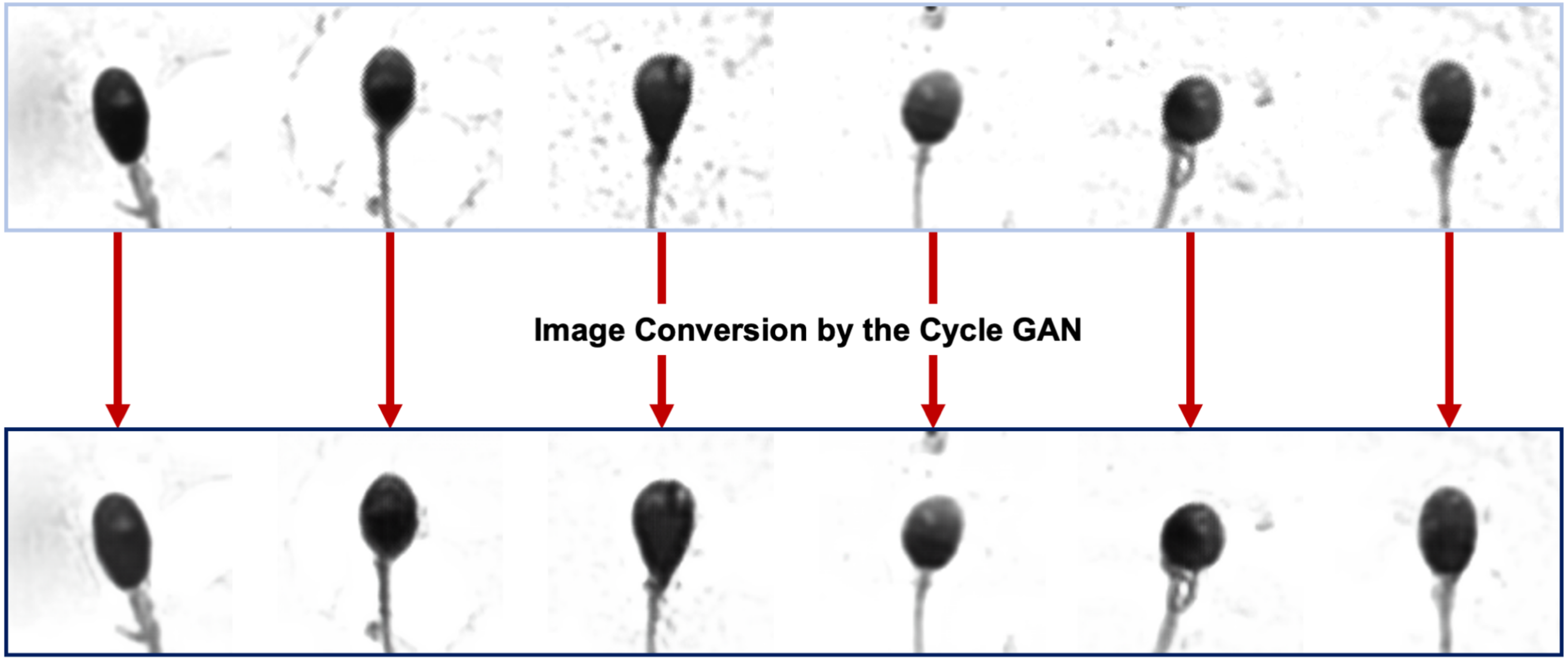

Supplementary Figure S3. Representative sperm images converted by the Cycle GAN model. The conditions under which samples were collected could lead to minor microenvironmental variations that were not visible to the naked eye, but might significantly confuse the deep learning classifier, causing misinterpretation of the datasets during training and validation. The Cycle GAN model collectively improved the overall image quality, such as removing background noise and enhancing outlines of the sperm heads. To ensure consistent image quality that accurately represented sperm morphology, the images of extracted sperm heads from clinical samples were converted to match the microenvironment of the laboratory samples. Cycle GAN: Cycle Generative Adversarial Network.

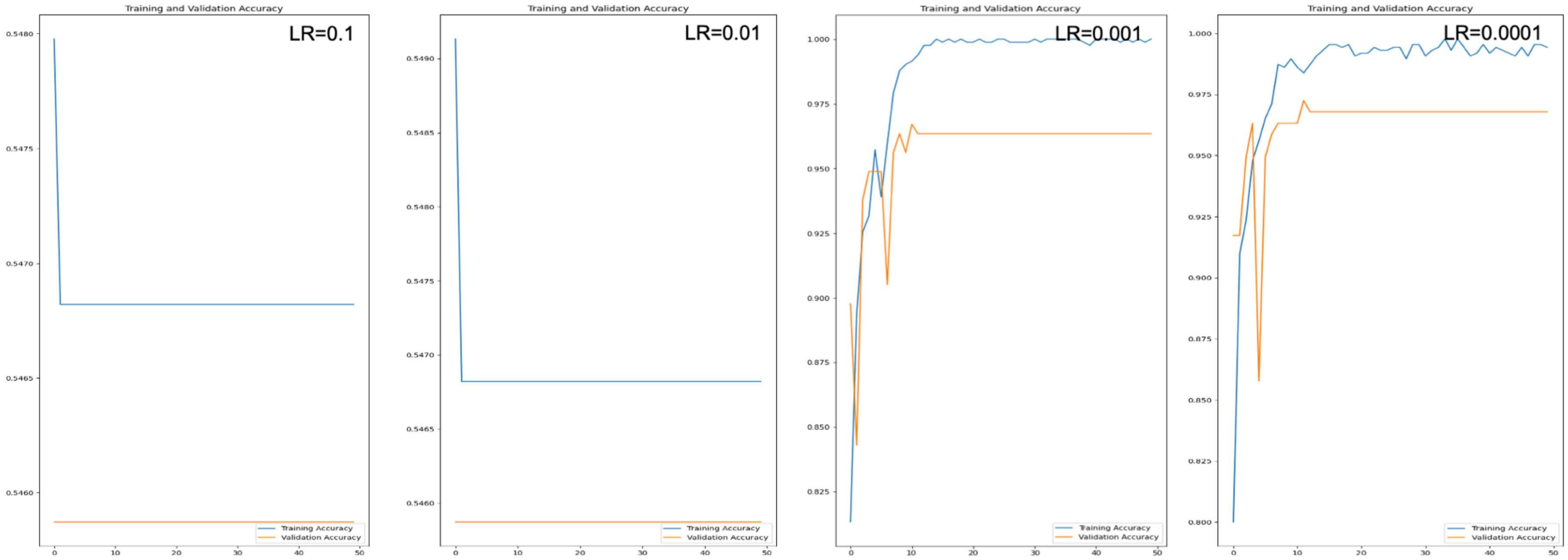

Supplementary Figure S4. Comparison of training accuracy of VGG13 at different learning rates. An optimal learning rate can greatly improve the overall training accuracy of the model. By finding the right balance between the learning rate and accuracy, the model can better negative its loss rates by adjusting the hyperparameters, which allows better generalization ability to unseen data. Our results demonstrated that the training accuracy of VGG13 deteriorated from over 90% to below 55%, with increasing learning rates (0.1 and 0.01), indicating the lack convergence to optimal condition during training. Thus, the learning rate of 0.001 was selected as the optimal parameter for our model.

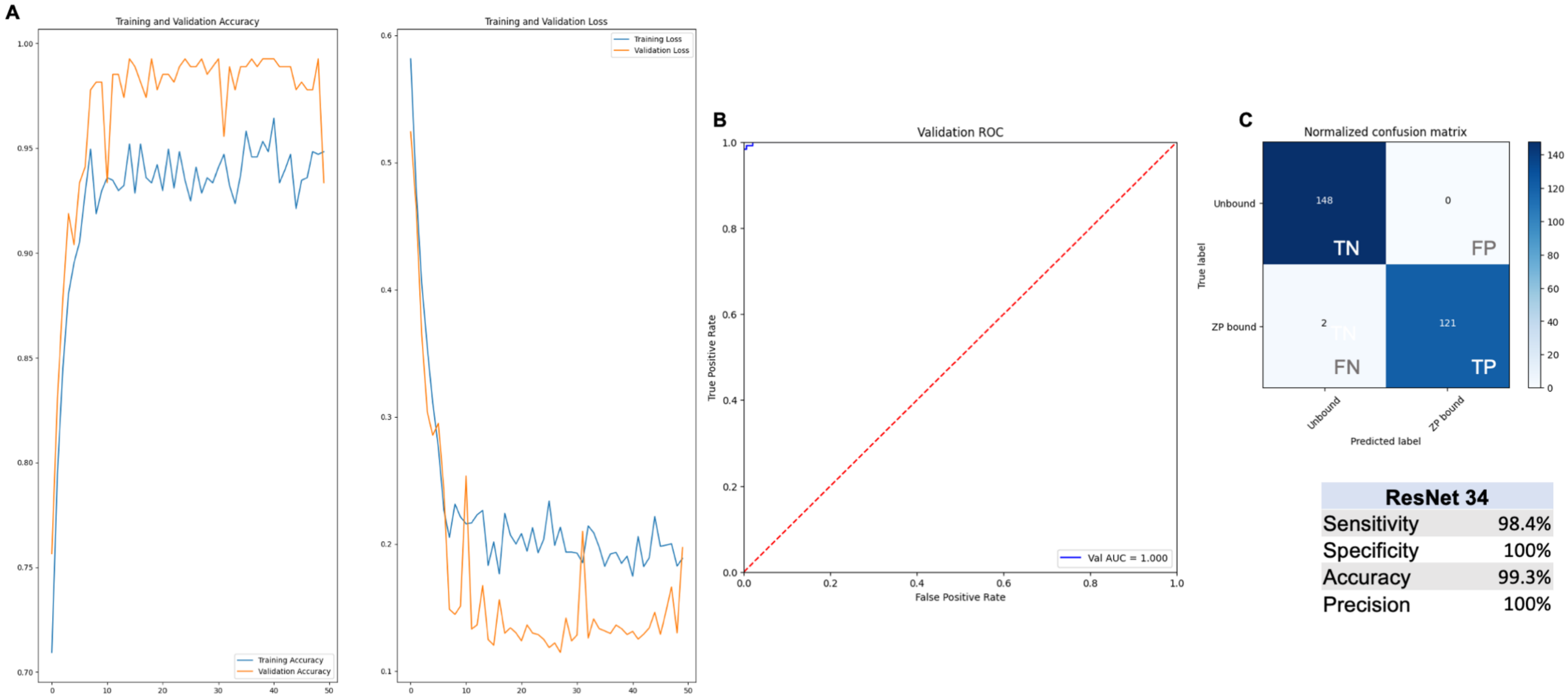

ResNet 34	
Sensitivity	98.4%
Specificity	100%
Accuracy	99.3%
Precision	100%

Supplementary Figure S5. Development of a ResNet 34 model to evaluate the ZP-binding ability of human spermatozoa. The ResNet 34 model was trained on our datasets using a batch size of 6 over 50 epochs with a learning rate of 0.00001 . **(A)** Accuracy curve: to evaluate the overall reliability of the model (Left). Cost curve: to measure the error rates of classifications (Right). **(B)** ROC curve : to examine the discriminative power of the model as reflected by the AUC value. **(C)** Confusion matrix: to examine the classification performance of the model for binary classification. The model demonstrated a good overall classification performance (sensitivity: 98.4%, specificity: 100%, accuracy: 99.3% and precision: 100%) for binary classification of human spermatozoa.

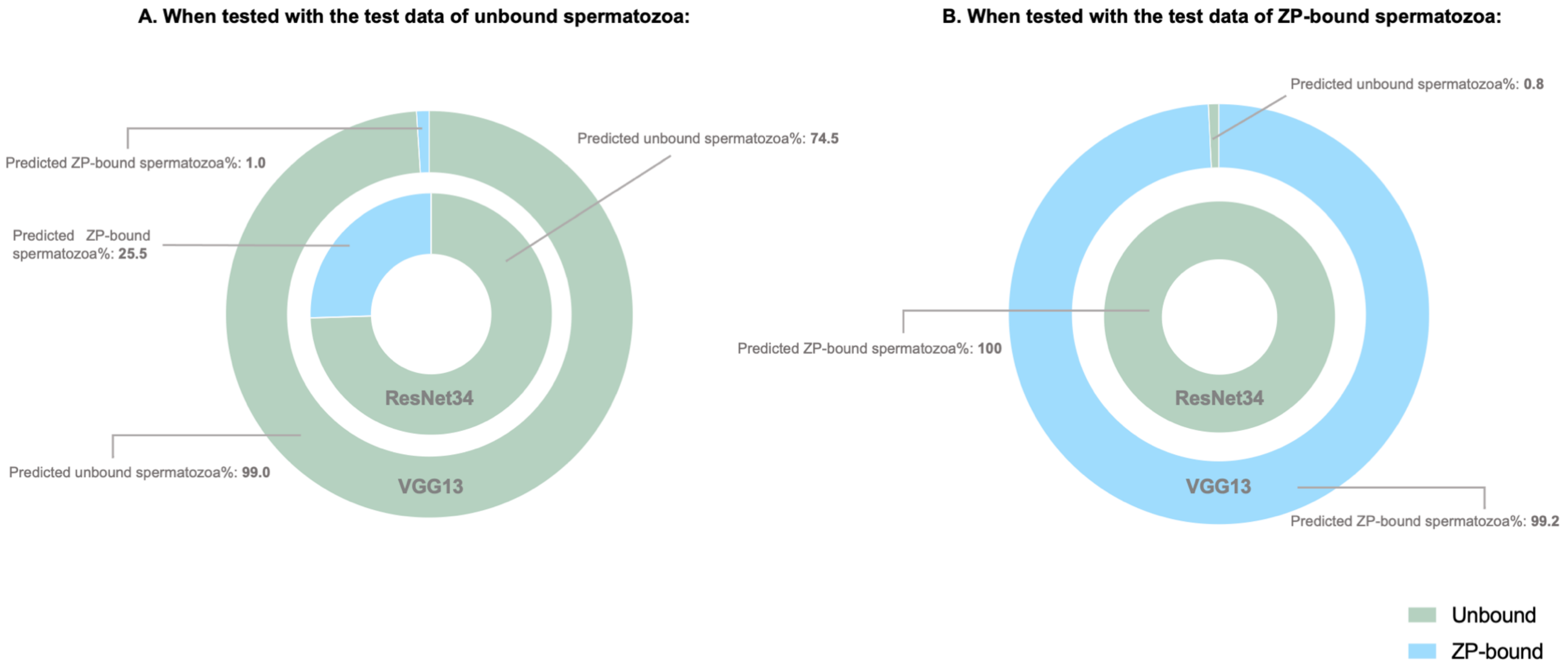

Supplementary Figure S6. Evaluation of generalization ability of the ResNet 34 and the fine-tuned VGG 13 models. (A and B) To further examine the generalization ability of the ResNet 34 and our fine-tuned VGG 13 model, both models were individually tested on two independent sets of test data of ZP-bound (n=122) and unbound spermatozoa (n=98) for binary prediction. **(A and B)** While the ResNet-34 model achieved a perfect 100% classification rate on the ZP-bound sperm dataset, it notably misclassified a significant number of spermatozoa in the unbound sperm dataset as having ZP-binding ability compared to the VGG13 model. These results suggested a potential issue of overfitting, leading to suboptimal generalization ability on unseen data.

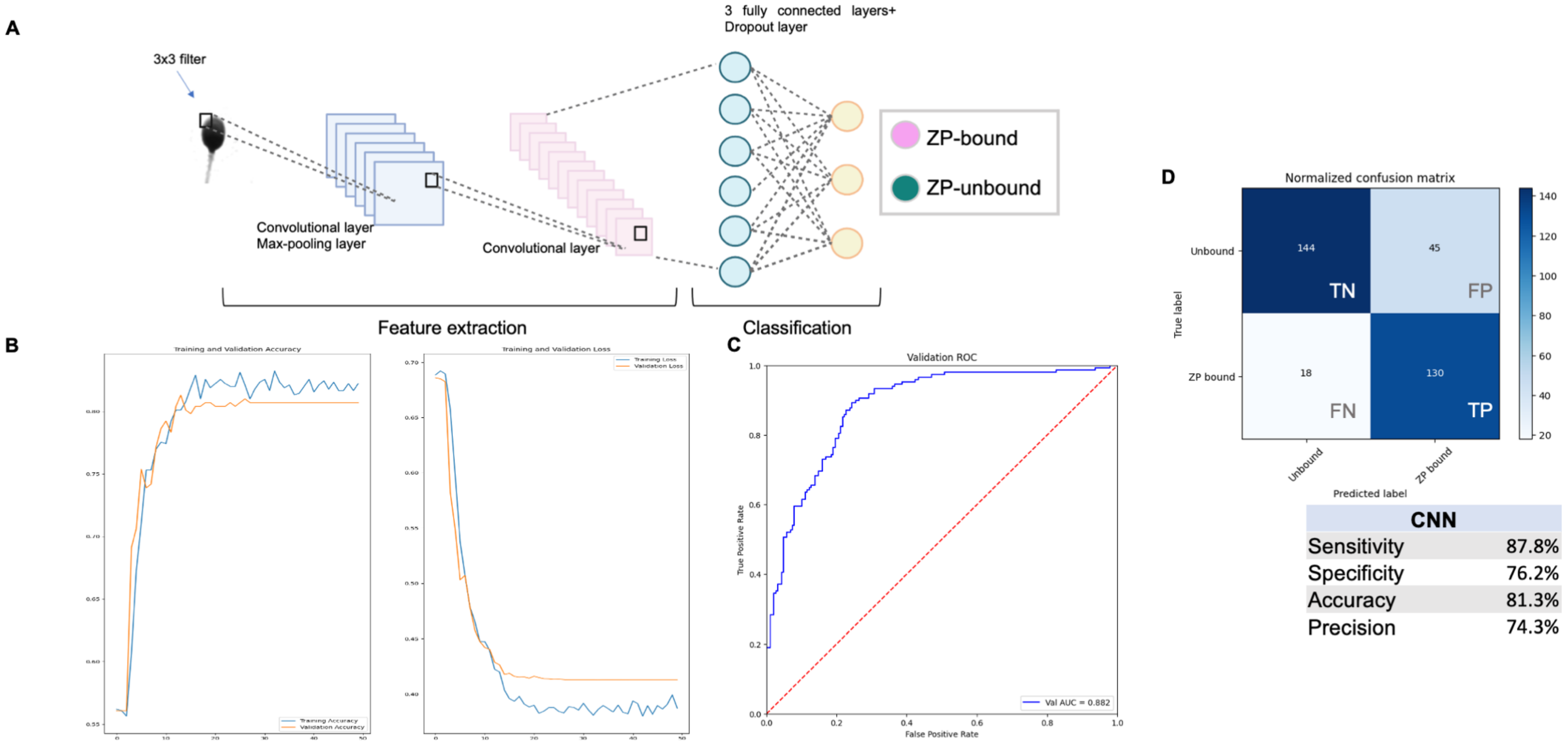

Supplementary Figure S7. Development of a basic CNN-based method to evaluate the ZP-binding ability of human spermatozoa. The basic CNN model comprised of 2 convolutional layers and 1 max pooling layers . It was trained on our datasets using a batch size of 6 over 50 epochs with a learning rate of 0.01 . **(A)** The images were inputted into the CNN model for binary classification **(B)** Accuracy curve: to evaluate the overall reliability of the model (Left). Cost curve: to measure the error rates of classifications (Right). **(C)** ROC curve : to examine the discriminative power of the model as reflected by the AUC value. **(D)** Confusion matrix: to examine the classification performance of the model for binary classification. Although the model was able to differentiate sperm subpopulations to some extent, its overall classification performance (sensitivity: 87.8%, specificity: 76.2%, accuracy: 81.3% and precision: 74.3%) was inferior to our fine-tuned VGG 13 model.

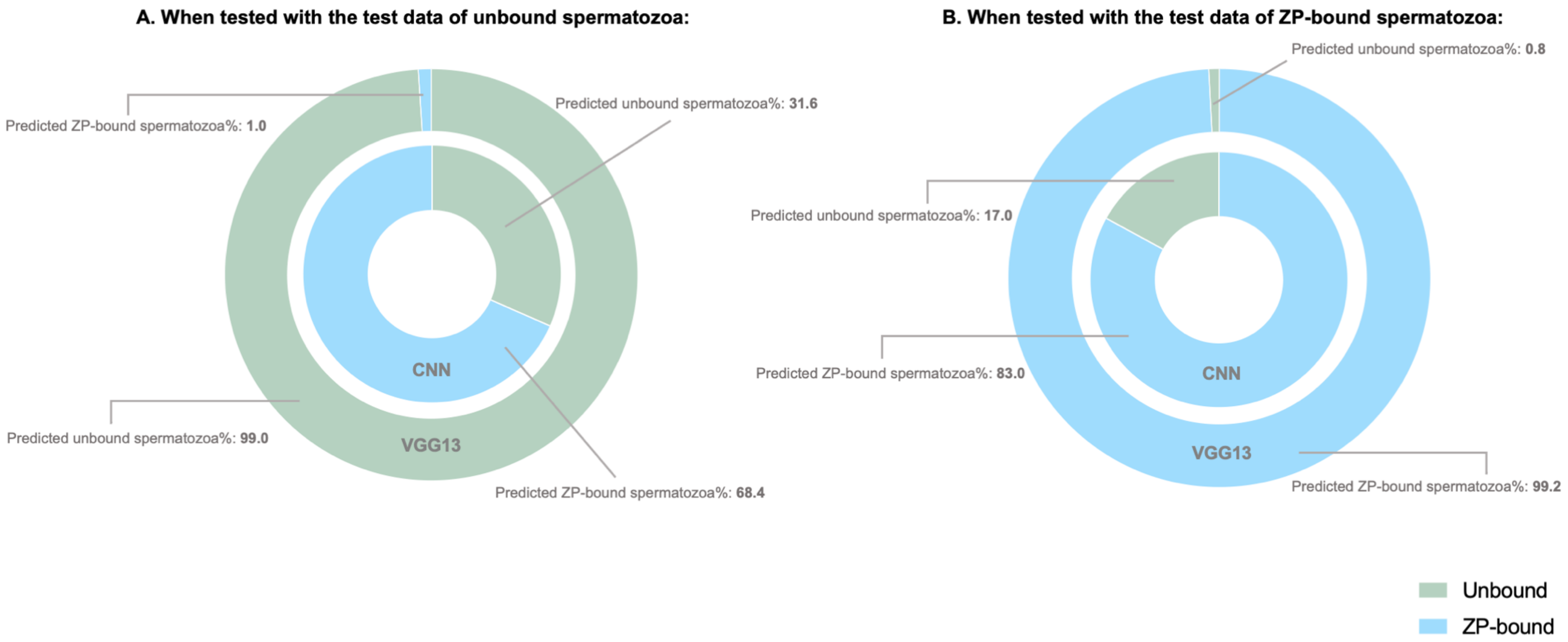

Supplementary Figure S8. Evaluation of generalization ability of the basic CNN and the fine-tuned VGG 13 models. (A and B) To further examine the generalization ability of the CNN and our fine-tuned VGG 13 model, both models were individually tested on two independent sets of test data of ZP-bound (n=122) and unbound spermatozoa (n=98) for binary prediction. **(B)** The basic CNN model misclassified a large proportion of spermatozoa in the unbound sperm dataset as the ones with ZP-binding ability, indicating its lack of good generation ability to unseen data for prediction.

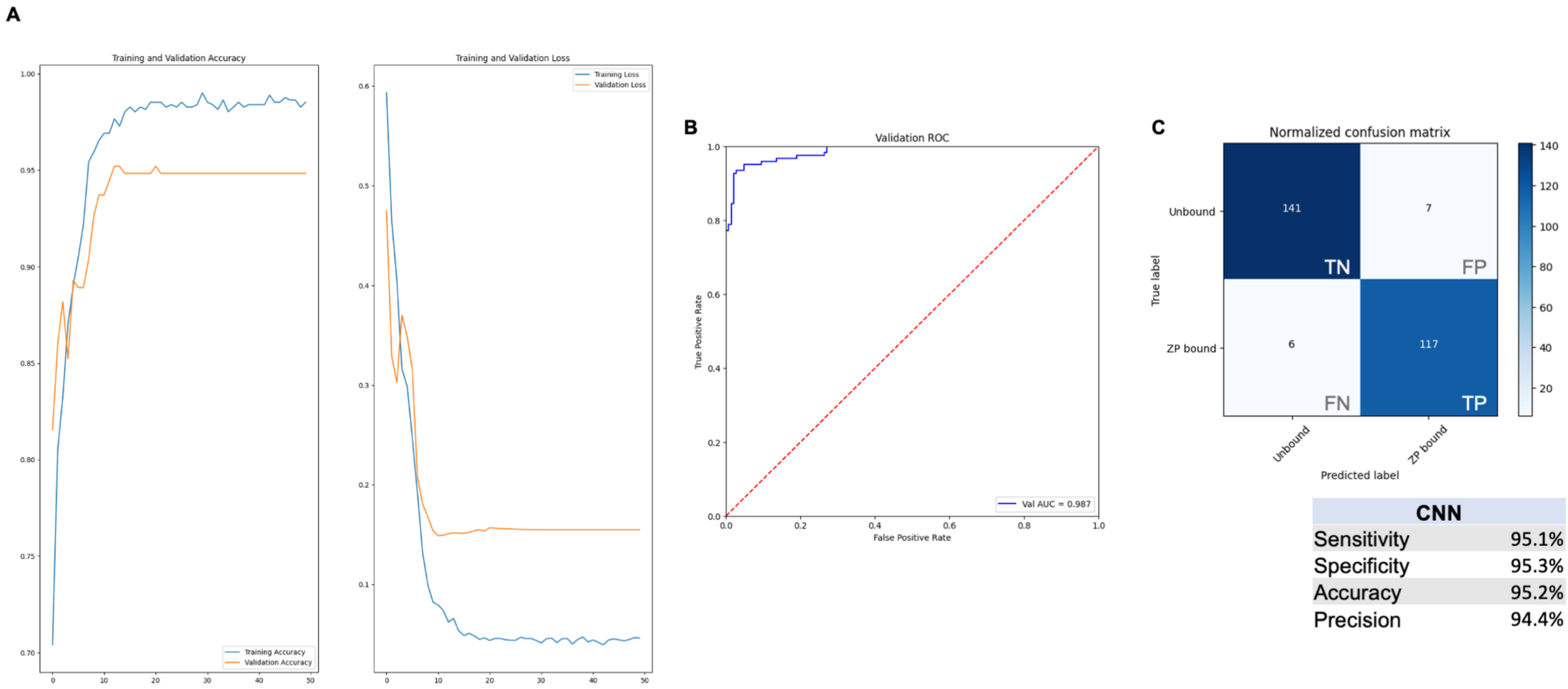

CNN	
Sensitivity	95.1%
Specificity	95.3%
Accuracy	95.2%
Precision	94.4%

Supplementary Figure S9. Development of a VGG 13 model trained on datasets of ZP-bound and unbound spermatozoa (without being subjected to GAN image conversion). The model was trained on our datasets using a batch size of 4 over 50 epochs with a learning rate of 0.01 . **(A)** Accuracy curve: to evaluate the overall reliability of the model (Left). Cost curve: to measure the error rates of classifications (Right). **(B)** ROC curve : to examine the discriminative power of the model as reflected by the AUC value. **(C)** Confusion matrix: to examine the classification performance of the model for binary classification.

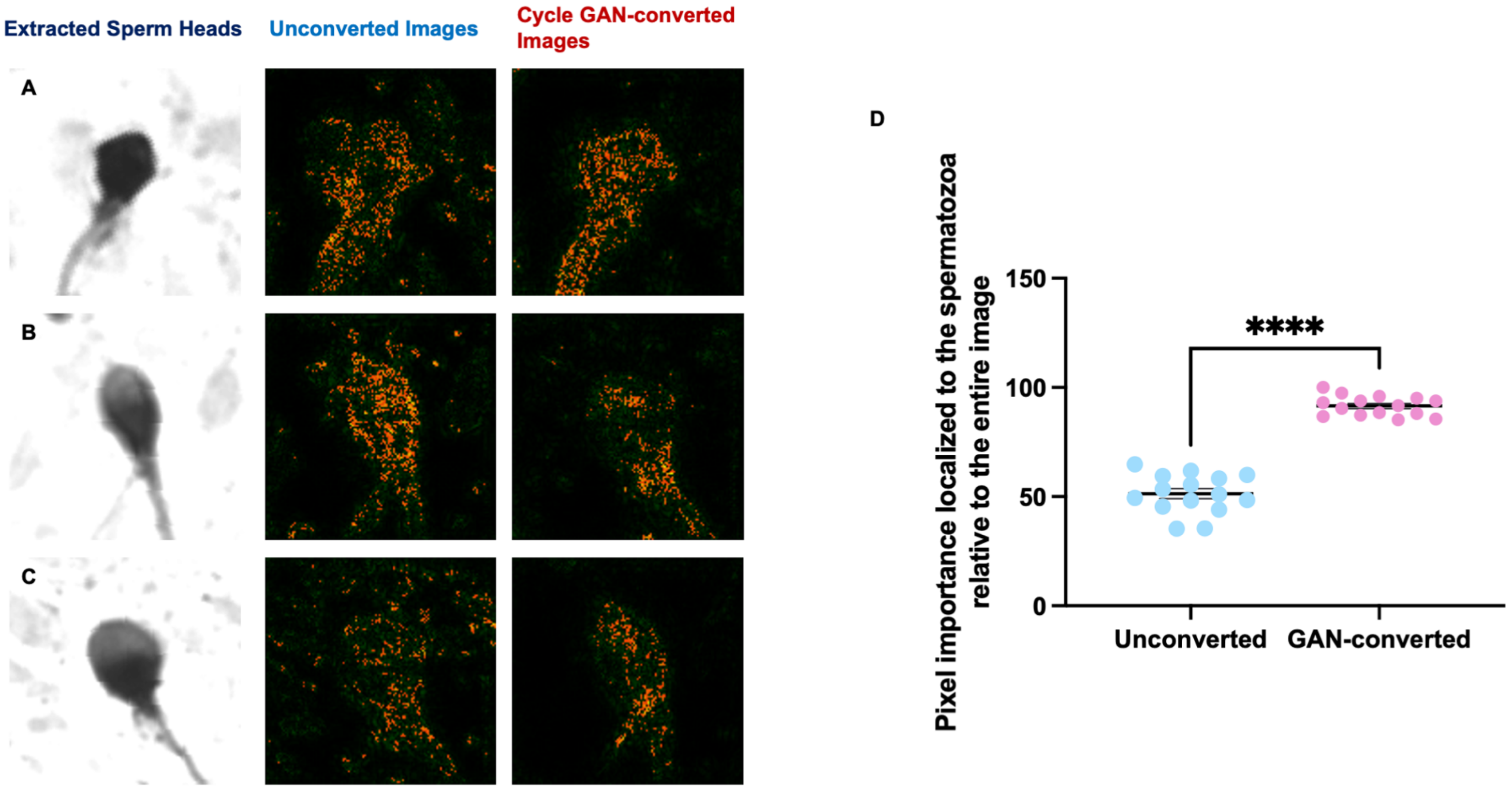

Supplementary Figure S10. Saliency maps of pixel importance between unconverted and cycle GAN-converted images. (A-B). Representative images of sperm heads before and after image conversion using the cycle GAN model**.**The saliency map overlay highlighted the relevant features that contributed to the prediction output of the model, as shown by the pixel importance in orange. **(D)** Qualitative analysis of pixel importance localized solely to spermatozoa relative to the entire region (n=15; P<0.05). In cycle GAN-converted images, approximately 91.6% of pixel importance clustered over the spermatozoa compared to approximately 51.4% in the unconverted images (with 48.6% focusing on the irrelevant components). All data are represented as mean ± SEM. CycleGAN: Cycle Generative Adversarial Network.
